# Research Progress of Peach Bacterial Spot Disease

**DOI:** 10.3390/ijms27062639

**Published:** 2026-03-13

**Authors:** Wenqing Lu, Wenxiao Du, Changlong Chen

**Affiliations:** 1 College of Life Sciences, Yantai University, No. 1 Siping Road, Yantai 264006, China; luwenqing0305@163.com; 2 Institute of Biotechnology, Beijing Academy of Agriculture and Forestry Sciences, Beijing 100097, China

**Keywords:** peach bacterial spot disease, *Xanthomonas arboricola* pv. *pruni*, biological control, plant resistance

## Abstract

Bacterial spot disease in peaches, also known as bacterial shot hole disease, with *Xanthomonas arboricola* pv. *pruni* (*Xap*) as the causal agent, poses a significant threat to peach yield and quality due to its long latency period, rapid onset, and difficulty in control. This article presents the first systematic review of research on the peach bacterial spot pathogen, *Xap*, comprehensively integrating the latest advances in disease distribution, pathogen identification, integrated control strategies, and mechanisms of pathogenesis and host resistance, thereby forming a complete and up-to-date knowledge framework. The aim is to provide a reference for the control of peach bacterial spot disease and to promote the sustainable and rapid development of the peach industry.

## 1. Introduction

Peach (*Prunus persica* (L.) Batsch) belongs to genus *Prunus,* subfamily *Amygdaloidae* of *Rosaceae*, and is a fruit crop with high ornamental and economic value that is cultivated worldwide. According to the Food and Agriculture Organization (FAO), the worldwide total harvested area for peaches and nectarines reached 1.56 million hectares in 2024, with a production output of 27.89 million tons. Compared to 2020, the harvested area increased by approximately 3.5%, while production increased by 14.1% [1]. As the area of peach cultivation expanded and varieties multiplied, the bacterial disease threat began to emerge. The bacterial spot of peach is mainly caused by *Xanthomonas arboricola* pv. *pruni* (*Xap*) [2]. The disease causes lesions on leaves, twigs, fruits and stem cankers, which will bring trouble to the fruit farmers, and seriously affect the yield quality and crop economy of the peach [3]. *X. arboricola* is listed as a quarantine organism in the European Union phytosanitary legislation (EU Directive 2000/29/CE) and in the European and Mediterranean Plant Protection Organization (EPPO A2 list) lists [4]. *X. arboricola* pv. *pruni* causes bacterial spot disease on stone fruits (including almond) and on walnut. Besides cultivated species, such as plum, peach and nectarines, and their hybrids, all the ornamental *Prunus* species can be affected by this bacterium [4]. Currently, chemical agents used for controlling bacterial spot disease in peaches tend to favor copper-based formulations, but long-term use is not only unfriendly to the environment but also causes the pathogen to develop resistance [3]. This review systematically integrates current advances in the distribution, pathogen identification, integrated control strategies, and pathogenesis of peach bacterial spot disease, offering a comprehensive reference and forward-looking perspectives for future research and disease management.

## 2. Distribution and Occurrence of Peach Bacterial Spot Disease Especially in China

**Figure 1:**
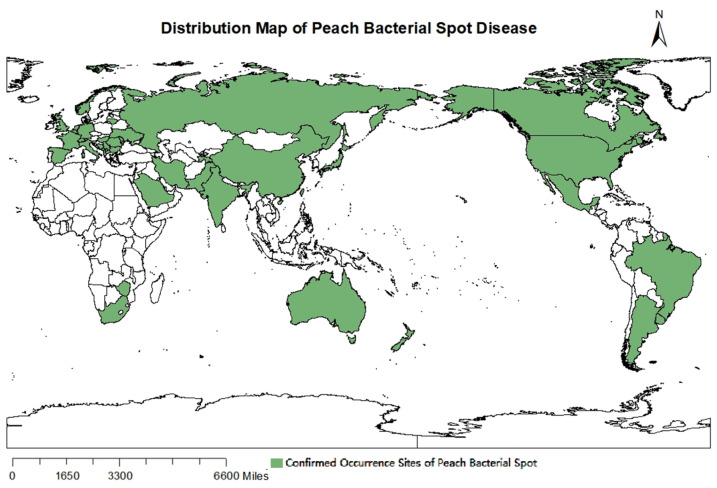
Documented global distribution of peach bacterial spots. It is supported by references [3,5,6,7,8].

The global peach industry faces serious threats from bacterial spot disease, which leads to substantial losses in both yield and fruit quality. This disease is distributed in 44 countries around the world, encompassing regions such as China, Canada, the United States, Japan, South Korea, Europe, and Africa (Figure 1) [3,5,6,7,8]. The severity of the disease varies significantly across these different geographical locations, and it is highly contagious, contributing to its widespread concern within the agricultural community [3].

**Figure 2:**
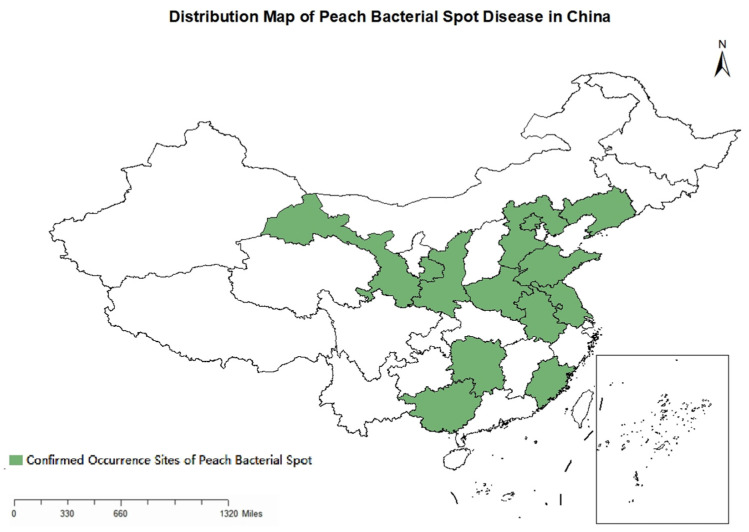
Documented distribution of peach bacterial spot disease in China. The box in the lower right corner indicates the South China Sea Islands. It is supported by references [11,12,13,14,15,16,17,18,19,20,21,22].

The causal agent of peach bacterial spot was first isolated in the United States in 1903 from plums [9]. However, it was not until the late 1970s that the specific pathogen, *X. arboricola* pv. *pruni* (*Xap*), was first identified in China, where researchers in Liaoning Province successfully isolated it from ‘XiangJiaoli’ plum trees [10]. Subsequent research and surveys have revealed its extensive dissemination. The disease has now been documented in 12 provinces and municipalities across the country (Figure 2), including Gansu, Shaanxi, Hebei, Beijing, Liaoning, Henan, Shandong, Anhui, Jiangsu, Hunan, Guangxi Zhuang Autonomous Region, and Fujian Province [11,12,13,14,15,16,17,18,19,20,21,22]. Given China’s vast territory and diverse climatic conditions, the occurrence of peach bacterial spot disease also varies considerably. For instance, in the Guilin area of Guangxi Province, the hot and humid climate is highly conducive to the disease. Symptoms first appear in mid-to-late March, with the peak incidence occurring during mid-April to mid-June, and the disease stops developing in August [21]. However, in Dalian, Liaoning Province, bacterial spot disease emerges with rising temperatures in April, with slow development continuing through May. The disease starts to become more severe in June, with average temperatures climbing above 20 °C and monthly precipitation around 50–100 mm. The epidemic culminates in a peak period from June to July, with the disease index starting to decrease from mid-to-late July [15]. This pattern of disease development could be closely correlated with local climatic conditions and the amount of rainfall during the growing season.

## 3. Symptomatology of Peach Bacterial Spot

**Figure 3:**
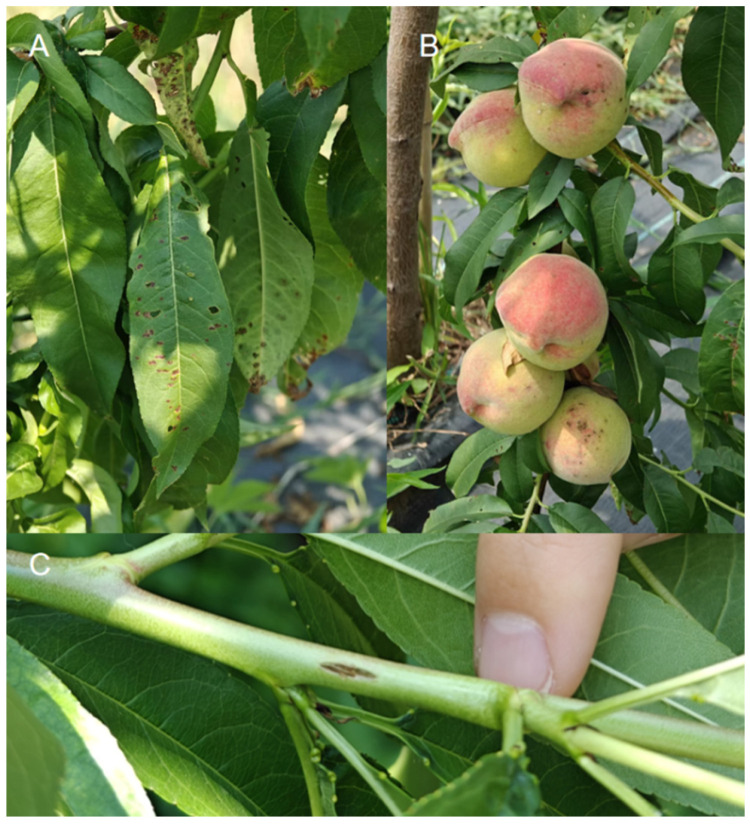
Typical symptoms of peach bacterial spots caused by *Xap* on different tissues. (**A**) Water-soaked lesions with chlorotic halos on leaves; (**B**) Sunken, necrotic lesions on fruit; (**C**) Cankerous lesions on a twig.

Infection by *Xap* can affect leaves, fruits, and twigs of peach trees, with characteristic symptoms developing on each organ (Figure 3). The symptoms of bacterial spot disease manifest differently across tree tissues. On peach leaves, infection begins with water-soaked spots on either side of the veins. These lesions subsequently enlarge, forming a distinct dark brown center encircled by a yellow halo. Ultimately, the necrotic centers may drop out, creating shot holes. On peach fruit, the initial symptoms are similar to those on foliage, but the lesions later become sunken and necrotic, drastically reducing marketability. On peach twigs, the disease presents as deep brown, cankerous lesions on the bark [4,16,23,24,25].

## 4. Pathogenic Mechanisms of *Xap*

*X. arboricola* pv. *pruni* (*Xap*) is the causal agent of bacterial spot disease, a devastating disease affecting peach production worldwide. This pathogen poses a significant threat to orchard yields and fruit quality, making understanding its disease cycle and pathogenesis crucial for developing effective control strategies. The pathogen survives during the winter latently in branches, buds, and leaf scars, resuming activity the following spring [3]. The development of the peach bacterial spot disease is affected by ambient temperature and humidity. Morales et al. [26] quantified this relationship, identifying 28.9 °C as the optimal temperature for infection. They demonstrated that to cause high disease severity, leaf wetness periods of at least 10 h were required at temperatures close to 20 °C, or 5 h were needed at temperatures between 25 and 35 °C.

Dispersal of *Xap* occurs via rain, wind, and possibly insect vectors, enabling the bacteria to reach new infection sites [27]. Upon arrival, a coordinated arsenal of processes is initiated including pathogen surface adhesion, biofilm formation, environmental sensing, chemotactic movement, etc. During disease development, cell-wall-degrading enzymes (CWDEs), effectors that inhibit plant immunity, and other virulence factors may contribute to bacteria proliferation [3]. Protective biofilms form on the plant surface in early stages of invasion shielding the bacteria from environmental stresses via their exopolysaccharide (EPS) matrix [3,28]. Early infection stages also involve environmental sensing mechanisms. This crucial step involves TonB-dependent transporters (TBDTs), two-component system sensors (STCRs), and methyl-accepting chemotaxis proteins (MCPs) [3]. They could be putatively associated with the adaptation of pathogens to their hosts [3,29]. During the infection process of the pathogenic bacterium *Xap*, the Type III Secretion System (T3SS) and the Type II Secretion System (T2SS) serve as the core apparatus for executing its virulence [24]. The T2SS is primarily responsible for secreting a suite of CWDEs into the extracellular environment. *Xap* possesses a series of genes encoding enzymes with cellulolytic, hemicellulolytic, and pectinolytic activities, which looks like it relies on those enzymes to break down the plant cell wall to provide nutrients for itself [3,29]. In contrast, the T3SS plays the more critical role of a “molecular syringe,” capable of directly injecting a class of virulence proteins known as Type III Effectors (T3Es) into the interior of host cells. The core function of these effector proteins is to acquire nutrients, promote infection, or evade immune responses by modulating host physiological processes, paving the way for successful bacterial colonization by suppressing the host’s defense responses [30]. The difference in T3SS and T3Es between pathogenic and non-pathogenic *X. arboricola* strains is extremely significant [31,32]. The former not only possesses a complete set of structural genes for the T3SS but also carries a rich and diverse repertoire of T3Es. In contrast, non-pathogenic strains generally lack key T3SS components and most effector proteins [33]. Furthermore, the virulence of *Xap* is also related to the plasmid pXap41 which carries important T3E genes and other virulence factors [24,31].

In recent years, research based on genomic comparisons has greatly enhanced our understanding of the Type III Secretion System (T3SS) of *Xap*. We can functionally categorize its Type III Effectors (T3Es) (Table 1), which provides a framework for understanding the hierarchical nature of its pathogenic strategy. As shown in Table 1, the effectors of *Xap* can be divided into three functional tiers: (1) The specific effectors including xopE3, xopAQ, xopAT and xopBA located on the plasmid pXap41 are the key determinants that distinguish *Xap* from other pathovars. During infection, the T3SS accessory secretion protein mltB aids these effectors in better infecting the plant [29,33,34,35,36]. These specific effectors act like “specialized keys” for successful colonization and pathogenesis in the specific host (peach). (2) The core effectors avrBs2, xopF1, xopA, xopR, along with two other accessory secretion proteins hpaA and hrpW, constitute the conserved core effector repertoire within the *Xanthomonas* genus. They are primarily responsible for suppressing plant basal immunity (pathogen-associated molecular pattern (PAMP)-triggered immunity, PTI), establishing a “basic environment” for infection [29,31,33,35]. (3) The accessory/expanded effector repertoire: This group varies in distribution among different strains and pathovars of *Xap*. Their functions are diverse and may synergistically or additively interfere with different host signaling pathways to enhance virulence and counter a broader range of host defenses [29,31,33,36]. However, it must be pointed out that current understanding remains largely at the level of “sequence presence” and “homology-based prediction”, lacking functional validation. The specific molecular functions and host targets of effector proteins in peach trees are still not characterized. Future research should focus on deciphering the functions of these key effectors, particularly the *Xap*-specific effectors. This will not only reveal the molecular basis of host specificity but also provide precise targets for designing targeted disease control strategies, such as breeding resistant varieties or developing effector inhibitors. As revealed in studies on the pomegranate bacterial blight pathogen *X. axonopodis* pv. *punicae*, its XopN effector promotes infection by localizing to the plasma membrane and suppressing key immune responses, such as PAMP-triggered ROS burst [37], which provides a clear direction for future functional and target analysis of *Xap*-specific effectors in peach. Future research must shift from comparative genomics to in-depth plant molecular pathological validation. Only by completing this functional characterization can precise control strategies targeting key virulence steps be designed.

## 5. Isolation and Identification of the Pathogen

*X. arboricola* comprises a number of economically important fruit tree pathogens classified within different pathovars [38]. To date, nine validly described pathovars have been reported within this species distinguished by their host specificity and disease symptoms [33,38]. Among these, three pathovars are considered economically important worldwide: *X. arboricola* pv. *pruni*, the causal agent of bacterial spot of stone fruit; *X. arboricola* pv. *juglandis*, which causes walnut blight; and *X. arboricola* pv. *corylina*, responsible for bacterial blight of hazelnut [38]. Numerous studies have identified *X. arboricola* pv. *pruni* (*Xap*) as the primary causal agent of peach bacterial spot disease [19,25,39]. However, research has also identified other bacterial pathogens capable of inducing similar symptoms. For instance, *Pantoea agglomerans* and *P. ananatis* were reported as causative agents of this disease in Henan Province, China [40], while novel *Bacillus cereus* species complex were identified in Liaoning Province [41]. Despite these findings, *Xap* remains the most significant pathogen. It is a Gram-negative bacterium belonging to the genus *Xanthomonas* (*Xanthomonadaceae*) [30,42], with colonies typically exhibiting a distinctive pale yellow, glistening, mucoid, and convex morphology [19].

The identification of *Xap* relies on a number of methods, encompassing morphological observation, physiological and biochemical characterization, and molecular biological assays. Among molecular techniques, PCR is a cornerstone. Early work by Park et al. [43] developed specific primers (*Xap*F/*Xap*R) based on the *hrp* gene sequences, which amplify a 243 bp fragment specific to *Xap* and show potential for detecting the pathogen in plant materials from peach orchards. Pothier et al. [44] designed a duplex-PCR assay combining pathovar-specific primers (*Xap*Y17, designed using random amplified polymorphic DNA (RAPD)) and species-specific primers (*XarbQ*, based on the *qumA* gene), validating its sensitivity and specificity in symptomatic plant samples and offering the first molecular test for all *X. arboricola* pathovars. In the same year, Palacio-Bielsa et al. [45] developed a highly sensitive real-time TaqMan PCR protocol targeting a gene for a putative protein related to an ABC transporter ATP-binding system in *X. arboricola* pv. *pruni*, achieving detection within hours and a sensitivity of 10^2^ CFU/mL, surpassing conventional PCR and is suitable as a screening test for *X. arboricola* pv. *pruni* as well as symptomatic or asymptomatic plant material. Similarly, Ballard et al. [46] developed a real-time SYBR Green I Bio-PCR (biological and enzymatic polymerase chain reaction) protocol capable of detecting fewer than 10 CFU and 0.1 pg of DNA for the detection of *X. arboricola* pv. *pruni*.

In addition, Pothier et al. [34] sequenced the plasmid pXap41 from *Xap* and evaluated its potential as a target for pathovar-level detection using plasmid profiling and multiplex PCR. For rapid field diagnosis, López-Soriano et al. [47] designed a prototype of a lateral flow immunoassay (LFIA) using carbon nanoparticle-conjugated polyclonal antibodies which were assembled on nitrocellulose strips, providing a reliable screening tool for symptomatic plants. To enhance the precision of live *Xap* bacterial cell detection, the use of DNA-intercalating dyes combined with qPCR protocols promoting the quantification of only viable cells (v-qPCR) was developed [48]. In general, the dyes including ethidium monoazide (EMA), propidium monoazide (PMA), its improved version PMAxx™, or a mix of some of them can be used in v-qPCR. The chemicals can penetrate bacterial cells with damaged membranes and covalently bind to double-stranded DNA upon photoactivation, leading to bounded DNA unamplified and amplification of only viable cells [49,50]. Sabuquillo et al. [51] showed that the nucleic acid-binding dye PMAxx™ is the most effective dye for detecting viable *Xap* bacterial cells among PMA, a combination of PMA and EMA, and PMAxx™.

Isothermal amplification methods offer alternatives to PCR. Li et al. [52] developed a loop-mediated isothermal amplification (LAMP) assay targeting an ABC transporter ATP-binding protein, which demonstrated high sensitivity and specificity for detecting *Xap* in field samples. Beyond amplification-based methods, strain sequencing is a fundamental approach for pathogen identification. Common techniques include 16S rRNA/rDNA sequencing and 16S–23S ITS sequencing. For example, Zhao et al. [19] preliminarily identified isolates from Lianyungang orchards as *Xap* by comparing their 16S–23S ITS sequences with GenBank records and correlating the results with morphological characteristics. Similarly, Robe et al. [25] identified the pathogen in China through morphological features, PCR and sequencing targeting the 16S rRNA and ABC transporter ATP-binding protein genes, and pathogenicity tests. The utility of 16S rDNA sequencing was further demonstrated by Luo et al. [2], who identified 98 *Xanthomonas* strains from 12 Chinese provinces using this method, and by Sun et al. [14] in Beijing. In addition, advancing towards novel nucleic acid detection platforms, Luo et al. [53] established an RPA/Cas12a-based system targeting the *ftsX* gene, which allows for rapid (within 2 h), highly sensitive, and specific detection of *Xap* even using crude DNA extracts at 37 °C, presenting a practical tool for field diagnosis. Multilocus sequence analysis (MLSA), based on sequencing conserved housekeeping genes: *fusA* (elongation factor 4), *gapA* (glyceraldehyde-3-phosphate dehydrogenase A), *gltA* (citrate synthase), *gyrB* (DNA gyrase subunit B), *lepA* (elongation factor 4), *lacF* (PTS system lactose-specific EIIA component), *fyuA* (tonB-dependent receptor), *dnaK* (molecular chaperone DnaK) and *rpoD* (RNA polymerase sigma factor RpoD), serves as a key method for accurate identification and population genetic studies of *Xap* [8,54]. For example, MLSA was used to identify *Xap* as the causal agent of a new disease on peach in Montenegro based on the housekeeping genes *fyuA*, *rpoD*, and *gyrB* [8] and to determine *Xap* strains from the Western Balkans belonging to Haplotype I, showing homology with the European population [54]. Thus, MLSA provides a reliable approach not only for accurate pathogen identification but also for tracing strain origins and deciphering population evolutionary relationships. The advancement of these molecular detection technologies reflects a shift from precise laboratory identification to rapid field-based early warning. Traditional isolation and culture are the standard methods for pathogen identification [19], but it is time-consuming and labor-intensive. Conventional PCR and qPCR have achieved a leap from “detection” to “quantification” [43,44,45,46,48,51], becoming the cornerstone of laboratory research. In contrast, technologies such as LAMP, RPA/Cas12a, and LFIA focus on addressing the challenge of point-of-care diagnosis in the field [47,52,53]. The choice of method depends on the specific application scenario: conducting in-depth pathological research, performing regulatory identification of suspicious samples, or carrying out large-scale field monitoring during the early stages of disease onset. Table 2 provides a systematic comparison of the principles, sensitivity, advantages, and limitations of these methods, offering a clear technical roadmap for plant protection professionals to select the most suitable diagnostic strategy based on their needs. It is important to note that balancing detection sensitivity, operational simplicity, cost, and equipment dependency remains the core challenge for developing widely applicable field diagnostic tools in the future.

## 6. Current Status of Control Technologies

### 6.1. Agricultural Control

Cultivation Management. The incidence of disease in peach orchards is influenced by multiple factors, including regional seasonality, ambient temperature, relative humidity, orchard aeration, and hygiene conditions. Therefore, selecting planting sites with sufficient sunlight, good ventilation, efficient drainage, deep soil layers, elevated terrain, and protection from strong winds is essential. Since high atmospheric humidity can exacerbate disease severity, establishing an effective drainage system around orchards is critical [55]. Where feasible, rain-sheltered cultivation is recommended to enhance tree resistance [56]. In addition, intercropping has a certain preventive and control effect on crop diseases. To reduce the risk of infection, intercropping with susceptible species such as plum and apricot should be avoided [55]. From an ecological perspective, Li et al. [57] found that intercropping peach trees with cherry trees can effectively reduce the incidence and severity of bacterial spot.

Fertilization Use. Scientific fertilization practices should be adopted, emphasizing the application of organic fertilizers and avoiding excessive nitrogen [3,55,58].

Pruning Practices. Branches harboring pathogens serve as primary inoculum sources for bacterial spot. Pruning overlapping branches and burning those showing disease symptoms can help inhibit pathogen overwintering [3,55,58].

Orchard Sanitation. Maintaining orchard hygiene by promptly removing fallen leaves and implementing deep tillage can significantly reduce initial inoculum levels [27]. Saumya Yadav et al. [59] developed a convolutional neural network model using deep learning and image analysis that can identify and quantify bacterial spot disease from leaf images of infectious peach crops, achieving an accuracy of 98.75%. This approach can be integrated with drone technology for targeted monitoring of diseased leaves in orchards, thereby optimizing management efforts and saving labor and time.

During the transportation of seedlings and fruits, the application of antimicrobial packaging could be a promising physicochemical synergistic strategy for disease prevention. This approach is supported by research on antimicrobial packaging materials, such as antimicrobial assays on binary and ternary polymeric films based on polyvinyl alcohol (PVA), chitosan (CH) and lignin nanoparticles (LNP) showing an inhibitory effect on bacterial growth of *Xap* over time [60]. The study by Yang et al. [61] also demonstrated that lignin nanoparticles exhibit significant antibacterial efficacy against Gram-negative bacteria, including *X. arboricola* pv. *pruni*. If applied to packaging materials, they could effectively reduce cross-contamination during transport, holding direct application prospects for mitigating the risk of long-distance disease spread.

### 6.2. Chemical Control

Owing to their rapid efficacy and ease of application, chemical agents remain the primary approach for controlling bacterial spot disease in most orchards [3]. Current management strategies emphasize prevention, with routine chemical applications being carried out annually.

Copper-based formulations are the most commonly used chemical agents for controlling bacterial spot disease in peach orchards [3]. Before disease outbreaks and after the end of the growing season, mixtures of copper sulfate and Bordeaux mixture are primarily sprayed. During the growing season, to avoid phytotoxicity, it is necessary to select highly dispersed copper-based formulations with low concentrations, such as copper sulfate, copper hydroxide, oxine-copper, and thiodiazole copper, among others. In contrast, the concentration of Cu^2+^ can be increased after the end of the growing season and during the dormant period [3]. Another category includes various formulations with distinct mechanisms of action, making them potentially applicable during the growing season to inhibit disease spread. Among these, chlorobromoisocyanuric acid stands out due to its low toxicity, easy degradability, and systemic translocation property: when dissolved in water, it releases hypobromous acid and hypochlorous acid, exhibiting the ability to kill both bacteria and fungi, thus being highly suitable for the integrated management of bacterial spot disease [62]. Field trials have also confirmed that a 1500-fold dilution of 50% chlorobromoisocyanuric acid soluble powder can effectively suppress the disease [56]. Additional effective chemical options include bromothalonil, benziothiazolinone, zinc thiazole, tebuconazole, zineb, and mancozeb, among others [3,56,63].

However, the unregulated or excessive use of chemical agents poses significant risks, including the development of pathogen resistance and environmental contamination in orchards. For instance, copper-tolerant strains have been identified in orchards with a history of frequent copper-based treatments [5]. In addition, copper-based chemicals can inhibit fruit tree growth, thereby compromising the effectiveness of future control measures [56].

### 6.3. Biological Control

The long-term reliance on chemical control measures, while effective in the short term, carries well-documented risks such as the development of pathogen resistance and environmental pollution for a wide range of plant pathogens. This underscores the critical need to develop sustainable and environmentally friendly alternative strategies for managing peach bacterial spot [24]. Biological control, which mainly utilizes beneficial microorganisms or their metabolites and plant-derived substances to suppress pathogens and enhance plant health, presents a promising approach [64]. Current research on the biological control of *X. arboricola* pv. *pruni* (*Xap*), the causal agent of peach bacterial spot, can be categorized into several strategies.

First, antibiotics of biological origin, such as tetramycin, zhongshengmycin, and ningnanmycin, have demonstrated potent efficacy against *Xap* in field applications, with control effects exceeding 86% in some cases [62,65]. Furthermore, the use of antibiotic combinations, such as mixtures of zhongshengmycin with benziothiazolinone, oxytetracycline, and zinc thiazole, or the sequential application of 20% oxytetracycline aqueous solution, 1.26% Xinjunan acetate aqueous solutions, 0.3% tetramycin aqueous solution, and 50% chlorobromoisocyanuric acid soluble powder, has shown synergistic effects, achieving control efficacies over 80% without harming tree growth [21,66]. However, the emergence of antibiotic-resistant *Xap* strains in orchards with frequent oxytetracycline and streptomycin use highlights a major drawback of this strategy, necessitating prudent application [67,68]. Furthermore, it should be noted that the application of antibiotics in agriculture is restricted or banned in many countries due to concerns about antimicrobial resistance and environmental safety [3].

Second, the use of antagonistic microorganisms represents a safe and rational alternative. Studies have identified various bacteria and fungi with inhibitory activity against *Xap*. These include non-pathogenic strains of *X. campestris* (nXc), *Lactobacillus plantarum*, the rhizobacterium *Alcaligenes faecalis* (strain YZ19), the endophytic fungus *Aspergillus* sp. JYY-3, and the marine fungus *Penicillium griseofulvum* UL-Ce9, which produces the antibacterial metabolite toluquinol [6,69,70,71,72]. Plant-growth-promoting rhizobacteria (PGPR) and microorganisms from extreme environments also show potential, offering diverse mechanisms such as niche competition [73].

Third, plant-derived natural products and resistance inducers are gaining attention. Bioactive compounds isolated from plants, such as stilbenes from *Rheum tanguticum*, pharbitin from *Pharbitis nil* seeds, and cinnamon essential oil, exhibit significant antibacterial activity against *Xap* [74,75,76]. Additionally, novel molecules like glucohumates (humic/fulvic acids) have been shown to control *Xap* incidence, likely by activating plant defense responses [77]. Recent findings showed phenolic acid purification from *Bromus inermis* straw has a control effect on *Xap*, with the main component p-coumaric acid playing an important inhibiting role and outperforming oxytetracycline, which further underscores the potential of this direction [78].

In addition, further studies have explored induced immunity and synthetic peptide strategies. Foix et al. [79] demonstrated that topical application of endogenous peptides PpPep1 and PpPep2 in *P. persica* could mimic PAMP-triggered immunity (PTI), protecting plants against *Xap* infection at nanomolar concentrations. Similarly, Oliveras et al. [80] designed bifunctional peptide conjugates (e.g., Flg15-BP475) that exhibit direct antibacterial activity against *Xap* while stimulating host immune responses, offering a sustainable dual-action control strategy.

In conclusion, given the concerns of the disadvantages of drug resistance and environmental pollution caused by long-term use of chemical agents and aligning with the global shift towards high-quality, green agricultural production, biological control offers a sustainable path forward for managing peach bacterial spot disease.

### 6.4. Resistant Varieties

The deployment of resistant cultivars represents a very effective strategy for disease control [81]. Breeding efforts, such as hybridizing susceptible Japanese cultivars with resistant Brazilian selections, have demonstrated a viable pathway to develop new varieties combining resistance to bacterial spot with superior fruit quality [82]. Pathogenicity assessments on seven different peach cultivars have identified ‘Zhongtao 9’, ‘Zhongyou 16’, and ‘Chunxue’ as more resistant to *X. arboricola* pv. *pruni* (*Xap*) [27]. Furthermore, varieties like ‘Souvenirs’ and ‘Whitewater’, released by the University of Arkansas, are noted for their robust resistance [83,84]. However, the development of resistant cultivars is complicated by the multitude of factors influencing disease incidence. Therefore, integrating modern biotechnological tools is imperative to accelerate the breeding of high-quality, disease-resistant peach varieties.

## 7. The Mechanisms of Disease Resistance

Breeding durably resistant cultivars stands as the most economically and environmentally sustainable long-term solution to peach bacterial spot disease [81,85]. However, achieving this goal is fundamentally dependent on deciphering the plant’s inherent resistance mechanisms. This understanding is pivotal, as it moves beyond simply documenting disease symptoms to revealing the molecular and physiological basis of plant immunity, as comprehensively reviewed for the broader plant immune system by Ngou et al. [86]. Consequently, elucidating these defensive strategies extends beyond theoretical inquiry; it establishes an indispensable foundation for developing next-generation disease management technologies. Early genetic studies, such as linkage map construction and quantitative trait loci (QTL) mapping, have laid the foundation for identifying potential resistance genes in peach [87]. Transcriptome sequencing has become a key tool for elucidating defense mechanisms. Upon pathogen infection, peaches activate innate defense responses, and RNA-seq enables detailed profiling of transcriptomic changes, revealing genes involved in resistance—such as those encoding PAMP receptors and disease resistance genes including several RPM1-like and pathogenesis-related thaumatin encoding genes [88]. Gervasi et al. [89] used RNA-seq to compare transcriptome dynamics in resistant (‘Redkist’) and susceptible (‘JH Hale’) peach cultivars at 30 min, 1 h, and 3 h post-inoculation with *X. arboricola* pv. *pruni* (*Xap*). They observed delayed but stronger defense activation in the resistant genotype, along with differential expression kinetics and exon usage. Furthermore, candidate resistance genes mapping to QTLs for resistance to *Xap*—including WRKY-like, CRK-like, CuAO-like, and TIR-NBS-LRR-like genes were regulated only in the resistant cultivar.

More recently, Zhu et al. [90] integrated mRNA and miRNA profiling in resistant (‘Yanbao’) and susceptible (‘Yingzui’) cultivars following *Xap* challenge. They identified 58 miRNA-target pairs in the resistant variety versus 79 in the susceptible one, highlighting resistance-related genes such as *SPL6*, *TIFY6B*, and polyphenol oxidase gene (*PPO*, *Prupe.4G041800_v2.0.a1*), providing new insights into the post-transcriptional regulation of peach immunity.

## 8. Suggestions on Future Research and Control

While we have synthesized the existing knowledge on peach bacterial spot disease, it must be admitted that research on *Xap* remains in a relatively early stage compared to many well-established plant-pathogen systems, particularly in the areas of molecular interaction mechanisms and the translation of findings into applied technologies. This inadequacy in research directly hinders the development of efficient and sustainable control strategies. Therefore, this chapter will summarize the shortcomings of current studies and, focusing on several key fields, propose priority directions for future research along with comprehensive management strategies possessing long-term potential.

### 8.1. Unresolved Functions of Xap Effectors

Although Type III Secretion System effectors are known to be crucial for the pathogenicity of *Xap* [24,31], the specific functions of these effectors and their host targets within peach trees remain unknown. Current research on *Xap* predominantly remains at the genomic level [29,36,91]. Key unresolved questions include which effectors constitute the essential “core arsenal” for pathogenesis and how they work synergistically to suppress plant immunity. The lag in functional research hinders the development of precision breeding strategies based on effector recognition or the design of targeted compounds. Therefore, this review proposes that future research should prioritize the identification and functional characterization of key pathogenic effectors in *Xap* for application in disease resistance breeding.

### 8.2. Challenges to Sustainable Resistance

Current orchard management still relies heavily on copper-based compounds and antibiotics (e.g., oxytetracycline), which has led to the widespread emergence of resistant strains [5,67]. More critically, these resistant strains show no difference in overwintering survival capacity on peach trees compared to sensitive strains, meaning they can persist and become permanently established in the orchard ecosystem once they arise [92]. This highlights the fragility of existing control strategies. Concurrently, traditional breeding for disease resistance is a slow process. Although transcriptomic analyses of resistant peach varieties have been conducted, the key gene regulatory networks responsible for resistance have not been fully identified and validated [90]. Therefore, breeding for resistant cultivars remains a long-term and arduous endeavor.

### 8.3. The Long-Term Potential of Biological Control

Biological control is widely regarded as a pivotal direction for sustainable agriculture. The screening of antagonistic microorganisms represents a major research focus in this field. For instance, recent studies have demonstrated that *Bacillus velezensis* can achieve a field control efficacy of up to 66.6% against the disease [93]. However, despite these significant improvements in efficacy, current strategies remain confined to the paradigm of “introducing a single exogenous strain”, and their effectiveness is still constrained by complex field environments. Research by Chen et al. [94] revealed that the microbiome of healthy peach orchard soil possesses functionalities that suppress disease and promote plant growth. Therefore, only through a deepened understanding of the interactions among the biocontrol agent, the pathogen (*Xap*), the host plant (peach), and the environmental microbiome can a fundamental shift be achieved in biological control—transitioning from short-term intervention to long-term, sustainable management.

### 8.4. AI (Artificial Intelligence)-Driven Smart Plant Protection

AI-Driven Field Prediction and Monitoring. As mentioned earlier, the occurrence of peach bacterial spots is closely associated with local climate conditions. Therefore, by integrating modern technology with historical orchard disease data, machine learning algorithms can be employed to construct more accurate real-time predictive models for disease risk [3], facilitating a shift from passive response to active prevention. For early disease monitoring, models can be trained to identify the early, subtle symptomatic features of peach bacterial spots (such as water-soaked spots), enabling rapid, non-destructive monitoring across large field areas. For example, the PlantCaFo model developed by Jiang et al. [95] demonstrates the potential for high-accuracy disease identification using limited samples.

AI-Driven Targeted Drug Design. In the future, following a clear elucidation of the core molecular mechanisms of peach bacterial spot, AI can be leveraged to intelligently design and screen small molecules or peptide-based inhibitors that specifically interfere with the pathogen’s disease-causing processes. For instance, in recent research, Zhao et al. [96] successfully deciphered the core molecular mechanism of citrus resistance to Huanglongbing and utilized AI technology to screen for small peptides that can effectively control the disease.

Moving forward, it is essential to deeply integrate AI models with agronomic knowledge to provide scientific and technological support for the development of green and sustainable control strategies.

## 9. Conclusions and Prospectives

Peach bacterial spot disease, caused by *X. arboricola* pv. *pruni* (*Xap*), poses a significant challenge to global peach industry sustainability. Current reliance on chemical pesticides is unsustainable, necessitating a strategic shift towards integrated management. Future strategies should be underpinned by diverse pillars: AI-driven monitoring, biological control, genetic improvement and AI-driven drug development. Advancing these solutions requires a deeper molecular understanding of beneficial rhizosphere microbiomes, *Xap* pathogenesis and host defense mechanisms, as well as AI technologies. The integration of these insights is crucial for developing effective and sustainable control systems.

## Figures and Tables

**Table 1:** Evidence-based summary of key Type III Effectors in *Xanthomonas arboricola* pv. *pruni* (*Xap*).

Category	Effector(s)	Primary Function (Based on Homology and Prediction)
*Xap*-Specific Effectors	xopE3	Key *Xap*-specific virulence determinant; essential for pathogenicity on *Prunus* spp. [29,33,34,35,36].
	xopAQ	*Xap*-specific effector on plasmid pXap41; crucial for bacterial spot disease on *Prunus* [29,35].
	xopAT	*Xap*-specific; detected only in some strains; function uncharacterized [36].
	xopBA	Predominantly in *Xap*; potential role in host adaptation [36].
Core Effectors	avrBs2, xopF1, xopA, xopR	Ubiquitous virulence framework for *X. arboricola*; supports basal infection and PTI suppression. Strains possessing only this core set exhibit weak virulence and cannot cause typical disease on *Prunus* [29,31,33,35].
Accessory/Expanded Effectors	xopN, xopX, xopZ, xopQ, xopK, xopV, xopL, xopAI, avrXccA1, avrXccA2, xopAH, xopAF, xopG, xopAW	Variable expanded toolkit in *Xap* and related pathovars; diversified mechanisms to disrupt plant immunity and enhance virulence [29,31,33,36].

**Table 2:** Comparative analysis of diagnostic methods for *Xanthomonas arboricola* pv. *pruni* (*Xap*).

Diagnostic Method	Principle	Tested Materials	Advantages	Limitations	Applicability
Traditional Isolation and Culture [19]	culturing and phenotypic identification (colony morphology, biochemistry)	diseased peach leaves and fruits	confirms viability; enables pathogenicity tests	time-consuming (days to weeks); requires expertise; affected by competing microbes	strain isolation for research; koch’s postulates
Conventional PCR [43,44]	amplification of specific DNA fragments (e.g., *hrp*, *qumA*)	DNA (from culture or plant tissue)	specific; cost-effective	cannot quantify or distinguish live/dead cells; lower sensitivity than qPCR	laboratory screening of symptomatic samples
Real-time PCR (qPCR) [45]	real-time DNA amplification using TaqMan probes	DNA (from various samples)	high sensitivity (10^2^ CFU/mL); specificity; quantitative	high equipment cost; cannot distinguish live/dead cells	laboratory for high-precision diagnosis and research
Bio-PCR [46]	short enrichment culture and real-time PCR detection using SYBR Green	infected plant tissue	high sensitivity (<10 CFU); specificity; quantitative; avoiding copper as PCR inhibitor	adding a couple of days for culture step	laboratory detection of low-level or latent infections
Viability qPCR (v-qPCR) [48,51]	qPCR with dyes (e.g., PMAxx™) to inhibit dead-cell DNA amplification	DNA from viable cells (in tissue)	quantifies live bacteria (10^3^ CFU/mL); assesses treatment efficacy	complex workflow; may not fully exclude dead cell signals	laboratory evaluation of active infection/treatment
Loop-Mediated Isothermal Amplification (LAMP) [52]	isothermal DNA amplification with multiple primers	diseased peach leaves and fruit	high specificity (for *Xap* only); high sensitivity (1.8 ng/µL DNA); simple (no DNA pre-extraction); rapid (45 min)	complex primer design; high contamination risk	field detection in peach orchards
Lateral Flow Immunoassay (LFIA) [47]	immunochromatographic detection with antibody-coated strips	symptomatic leaf samples from infected peach trees	specific (detects *Xap* strains); rapid (10 min); simple and field-deployable; cost-effective	lower sensitivity (10^4^ CFU mL^−1^); potential false negatives	on-site field screening for symptomatic plants
Sequencing (16S/ITS, MLSA, Specific Genes) [2,8,19,25,54]	reading DNA sequences (universal or specific genes) for identification and analysis.	DNA (from pure bacterial culture)	definitive identification; high-resolution strain typing and phylogeny; enables epidemiological tracing	requires pure culture; time-consuming and expensive; laboratory-bound, not rapid	laboratory identification, strain characterization, and research.
RPA/Cas12a-based Detection [53]	RPA/Cas12a based-detection for visual (fluorescence/lateral flow assay)	infected peach leaf tissues	extremely high sensitivity (10^−18^ M or 1.63 copies/µL *Xap* gDNA for fluorescence assay and 10^−17^ M *Xap* gDNA for lateral flow assay); high specificity; rapid (~2 h) isothermal (37 °C)	fluorescence method may have background interference; lateral flow strips are less sensitive	on-site field detection

## Data Availability

No new data were created or analyzed in this study. Data sharing is not applicable to this article.
